# Indication for surgical treatment in patients with adolescent Idiopathic Scoliosis – a critical appraisal

**DOI:** 10.1186/1754-9493-7-17

**Published:** 2013-05-24

**Authors:** Hans-Rudolf Weiss, Marc Moramarco

**Affiliations:** 1 Orthopedic Rehabilitation Services, Gesundheitsforum Nahetal, Alzeyer Str. 23, Gensingen D-55457, Germany; 2 Scoliosis3DC, 3 Baldwin Green Common, Suite 204, Woburn, MA 01801, USA

**Keywords:** Scoliosis, Surgery, Complications, Indications

## Abstract

A recent literature search of the pertinent publications in the field revealed that there is poor evidence that would support surgical intervention in patients with Adolescent Idiopathic Scoliosis (AIS). With complications estimated to exceed 50% over a lifetime, surgical intervention is unwarranted in the ‘Adolescent Idiopathic Scoliosis’ AIS population. In the relatively benign population of patients with AIS, according to the findings in literature, we may conclude that the long-term outcome of surgery for AIS creates a more negative end result over the course of a lifetime than the natural history of the condition itself.

As a result, surgeons electing to recommend surgery are strongly advised to openly discuss and inform patients of the long-term probability of potential complications occurring after spinal fusion surgery, and document their explanations accordingly.

## Introduction

Scoliosis is a three dimensional deformity of the spine and trunk, which may deteriorate quickly during periods of rapid growth [1]. Although scoliosis may be an expression or a symptom of certain diseases, (eg. neuromuscular, congenital, due to certain syndromes or tumors), the majority of the patients with scoliosis (80 – 90%) are “idiopathic” because an underlying cause has not been determined [1]. The treatment of symptomatic scoliosis should be determined by the underlying cause, whereas, treatment of idiopathic scoliosis is determined by the deformity itself. Most scoliosis progresses during growth, some in later life; therefore, the main aim of any intervention should be to stop curvature progression [1].

While children grow until they have fully matured, there are certain times with more or less growth during childhood and adolescence. Curvature progression is probable during these different phases of growth [1].

In principle, the diagnosis of Adolescent Idiopathic Scoliosis (AIS) describes a spinal curvature in an otherwise healthy individual. According to the Scoliosis Research Society (SRS), the prevalence of AIS is 2% to 3% in the general population. Nearly 10% of AIS patients require some form of treatment and up to 0.1% will eventually require surgery [1]. AIS is more commonly found in females (female: male ratio 7:1) and usually AIS does not cause any health problems during growth.

Long-term follow-ups of untreated patients with AIS indicate that the consequences of AIS over a lifetime are minimal, sometimes moderate in more severe cases, but never life threatening. The curvature in this AIS population will usually not exceed 80 degrees. This degree of curvature will not affect vital capacity (VC) or breathing mechanics in the same way that other severe health conditions which compromise the cardio-pulmonary system may in the long term [1].

Treatment of AIS, when necessary, consists of physiotherapy, bracing, and spinal fusion surgery. While there is limited documented evidence, to date, for physiotherapy [1,2], there is a substantial body of evidence for bracing [1,3].

For spinal fusion surgery, no prospective controlled or randomized studies have been found [4,5]. Westrick and Ward [6] state: ‘No long-term, prospective controlled studies exist to support the hypothesis that surgical intervention for AIS is superior to natural history. Although surgery reliably arrests the progression of deformity, achieves permanent correction, and improves appearance, there is no medical necessity for surgery based on the current body of literature.’

Therefore, we may conclude from what is available in international literature that there is no medical necessity for surgery in the AIS population.

A long list of possible complications have been unveiled during the last decade [5,7,8] regarding AIS fusion surgery. Although the short-term complications may be minimal, long term complications have been estimated to exceed 50% with a rate of salvage surgeries of up to 25% [5,7,8]. Granted, these results were from populations treated with ‘older’ instrumentations such as Harrington rod or VDS procedures that are no longer applied today.

However, in consideration of the recently published paper containing the long-term results of the first ‘modern’ double rod instrumentation, the Cotrel-Debousset (CD) instrumentation, which revealed an unexpectedly high rate (nearly 50%) of reoperation due to late infections or chronic back pain [8] the argument opposing surgery is supported.

The last systematic review on long-term complications, as they might develop over a lifetime, was published in 2008 [7]. The first author conducted a Pub Med search to locate studies related to long term outcomes of AIS surgical complications published after August 2008 (publication date of the last review on complications [7]).

Two papers were found on the topic of reoperation rates with a long-term follow-up of at least 10 years [5]. Reoperation rates were between 12.9% and 47.5%.

Although the problem with post-surgical complications is highly relevant [5], reporting does not seem to be of utmost importance to surgeons, as evidenced by the lack of published literature. One drawback is that reporting complications is not mandatory [5]. Therefore, in literature we find a variety of non-standardized studies with various follow-up times inhibiting proper comparison.

Based on the experience of the authors, in Germany and the U.S., the majority of surgeons continue to inform otherwise healthy AIS patients (and their parents) that surgery is necessary at a given Cobb angle even when the patient is near or at bone maturity. However, for nearly all the AIS population there is no medical indication for such spinal fusion surgery. Signs and symptoms of scoliosis cannot be changed in the mid-term and cosmetic improvements initially achieved with surgery are not necessarily stable [5]. When we consider that the long-term course of AIS is far from being disastrous, we may conclude that the consequences of scoliosis surgery are far more dangerous and detrimental than the condition itself.

Although no paper on long-term complications was discovered when reviewing recent literature, it is worthwhile to look at the papers located in the search.

Low rates of complications (< 20%) have been reported in two papers on the long-term outcome of two different instrumentations and low back pain has been an issue in the population undergoing the CD instrumentation [5].

Other papers seem to reveal little rates of complication, and reoperation rates less than 10% [5]. Reoperation rates were between 3.9% at 2 year follow-up and 9.2% at 8.3 years follow-up. So, reoperation rates increase with time [5].

In conditions other than AIS, complication rates have been found to be rather high [5]. After less than three years a complication rate has been found between 26% (follow-up 14.3 months) and 42% (follow-up 28 months).

Obviously, back pain postoperatively is a problem [5] in the population of patients with AIS and disc degeneration, which increases after surgery [5]. Patients reported increasing post-surgical pain as time elapsed after AIS surgery [5].

A selection bias seems to exist in post-surgical studies [5]. The population “lost to follow-up” had more pain and less function in the specific SRS-22 domains than the “follow-up” populations in the studies. This most likely means patients with a worse outcome after surgery are more likely to seek advice with another healthcare provider and do not return to the surgeon who performed the initial operation. From this, two conclusions can be drawn:

1. The long-term outcome papers on surgical treatment are underestimating complication rates and/or failures.

2. Future studies on the outcome of surgery should add the “lost to follow-up” group to the failure/complication population.

Since there is no evidence indicating surgical correction in patients with AIS [4-6] and post-surgical complications are estimated around 50% over a lifetime [5,7,8], no claims can be made for a medical indication for such surgery. In the relatively benign population of AIS patients according to the findings within this review we may conclude that the long-term outcome of surgery for AIS is worse than the long-term consequences of the condition itself.

On the other hand, with bracing technology available today, a far more than 90% success rate can be expected in patient samples according to the SRS inclusion criteria for scoliosis bracing [9]. With the actual high correction braces a comfortable treatment can be expected leaving the patient pain free given that there is a professional providing brace adjustments with the appropriate skills. In this light, it seems questionable when new surgical techniques are applied which do not halt progression in curves exceeding 50° and no long-term experience is evident in the range of the general bracing indication, for curves between 25 and 41° [5].

There are numerous papers that base conclusions on quality of life questionnaires, mainly the SRS-22 claiming for a high patient satisfaction after scoliosis surgery [4]; however, the results or conclusions derived from these studies are questionable when considering the “dissonance” effect, in the paper referred to earlier, as it relates to post-surgical interviews [4]. Cognitive dissonance occurs most often in situations where an individual must choose between two incompatible beliefs or actions and there is a tendency for individuals to seek consistency among their cognitions. Unable to face an inconsistency, such as being dissatisfied with a surgical procedure, a person will often change his/her attitude. Surgery is impossible to reverse, but subjective beliefs and attitudes can be altered more easily. As a result, a patient not satisfied with a surgical procedure may not necessarily admit this [4].

Surgical intervention in AIS should only be considered in patients with substantial psychological problems due to the deformity. An indication for spinal fusion surgery may present itself when socialization is self-restricted because of the deformity. However, this is rarely the case in a population treated conservatively according to the latest standard. In a recent paper (with a sample of patients fulfilling the SRS inclusion criteria for studies on bracing) after completion of brace treatment no patient considered a surgical intervention [4].

However, in a patient requiring surgery, informed consent must be obtained for patient awareness and a surgeon’s liablility.The patients must be aware of the high percentage of long-term complications of fusion surgery and the amount of long-term complications to be expected [5,7,8]. Additionally, the stress the patient has due to the deformity must be documented. Therefore, the first author developed a brief questionnaire (BSSQ) in 2006 [5], which has been implemented and validated in several languages [5]. It is highly recommended to complete the preoperative patient awareness documentation of possible complications sometimes presenting greater than 20 years postoperatively [5,7,8]. This documentation in conjunction with the deformity related stress level questionnaire should be read carefully allowing full disclosure of long-term effects.

Currently, there are a few published studies comparing surgical to non-surgical treatment of adult scoliosis patients [5], however, none of these were with an untreated control group. Although these studies have limitations, the authors draw conclusions in spite of their major shortcomings. In some studies there were drop-out rates of greater than 50% in the non-operative group, so no conclusions are justified from these papers because a ‘worst case’ analysis would possibly lead to the opposite conclusions [5].

We are just at the start of evaluating the problems arising from spinal fusion surgery. The search for adverse effects of this kind of surgery is compromised by the fact that there is still no mandatory reporting of complications and implant failure [5]. As time passes, post-surgically more problems seem to emerge. This can only be seen as ‘the tip of the iceberg’ as many issues are not yet investigated simply because in later life various problems are not necessarily attributed to spinal fusion surgery [5].

Furthermore, in a recent article, ‘metallosis’ is described and it is stated that the consequences of the findings are not yet clear [cited in 5]. Cundy et al. state the following:

“A significant and rapid rise in serum titanium and niobium levels was observed within the first post-operative week, after which elevated serum levels persisted out to 12 months. Conclusions. We report abnormally elevated serum titanium and niobium levels in patients with titanium-based spinal instrumentation out to 12 months. The long-term systemic consequences of debris generated by wear and corrosion of spinal instrumentation is unclear but concerning, particularly as these implants inserted into the pediatric population may remain in-situ for beyond six decades.”

Recently a few critical reviews have been published clearly showing that (1) evidence for spinal fusion surgery is poor, (2) the longer the follow-up time the more complications are documented and (3) the clinical results after surgery do not seem better than the clinical results following conservative management of the latest standard [5,9].

Instead of devaluating conservative treatments [10] the surgical communitiy should focus on the long-term complications of spinal fusion surgery as conservative treatment is far from the disatrous long-term effects as described to impact patients after surgery for scoliosis.

## Conclusions

– A medical indication for spinal fusion surgery according to current literature is questionable.

– Complications of spinal fusion surgery in the long-term appear to exceed the possible limitations patients with AIS have to expect.

– Rate of complication of spinal fusion surgery appears to increase with time after surgery.

– There is poor evidence that spinal fusion surgery would improve signs and symptoms of AIS.

## Competing interest

HRW is advisor of Koob GmbH & Co KG, Abtweiler, Germany, MM declares to have no competing interest.

## Authors’ contributions

HRW: Literature review, draft of the manuscript, MM: Copyediting and additions to the manuscript as necessary. Both authors read and agreed to the content of the manuscrtipt as submitted.
